# A European Multi-Center Analysis of Extracorporeal Photopheresis as Therapy for Chronic Lung Allograft Dysfunction

**DOI:** 10.3389/ti.2023.11551

**Published:** 2024-01-12

**Authors:** Alberto Benazzo, Cecilia Bagnera, Fabio Ius, Claudia Del Fante, Jens Gottlieb, Konrad Hoetzenecker, Federica Meloni, Peter Jaksch, Mark Greer

**Affiliations:** ^1^ Department of Thoracic Surgery, Medical University of Vienna, Vienna, Austria; ^2^ Malattie dell’Apparato Respiratorio, Fondazione IRCCS Policlinico San Matteo, Pavia, Italy; ^3^ Department of Respiratory Medicine, Hannover Medical School, Hannover, Germany; ^4^ Department of Cardiothoracic, Transplantation and Vascular Surgery, Hannover Medical School, Hannover, Germany; ^5^ Servizio Immunoematologia e Medicina Trasfusionale, Fondazione IRCCS Policlinico San Matteo, Pavia, Italy; ^6^ German Centre for Lung Research, Biomedical Research in End-Stage and Obstructive Lung Disease Hannover, Hannover, Germany

**Keywords:** lung transplantation, extracorporeal photopheresis, CLAD, FEV1, lung function

## Abstract

Extracorporeal photopheresis (ECP) is used by few lung transplant centers to treat chronic lung allograft dysfunction (CLAD). Although reported results suggest a beneficial effect on CLAD progression, evidence is limited to single center experiences. The aim of this study is to analyze outcomes of ECP in a large multicenter European cohort. The primary endpoint was patient survival after initiation of ECP. This study included 631 patients, 87% suffered from bronchiolitis obliterans syndrome (BOS), and 13% had restrictive allograft syndrome (RAS). Long-term stabilization was achieved in 42%, improvement in 9%, and no response in 26%. Within the first 12 months of therapy, 23% of patients died. Patients’ survival after initiation of ECP at 5 years was 56% in stable, 70% in responders, and 35% in non-responders (*p* = 0.001). In multivariable Cox regression, both stabilization (HR: 0.48, CI: 0.27–0.86, *p* = 0.013) and response (HR: 0.11, CI: 0.04–0.35, *p* < 0.001) to ECP were associated with survival. Absolute FEV1 at baseline was also protective (HR: 0.09, CI: 0.01–0.94, *p* = 0.046). RAS phenotype was the only risk factor for mortality (HR: 2.11, 1.16–3.83, *p* = 0.006). This study provides long-term outcomes of ECP use in CLAD patients in the largest published cohort to date. Two-thirds of the cohort had a sustained response to ECP with excellent long-term results.

## Graphical Abstract

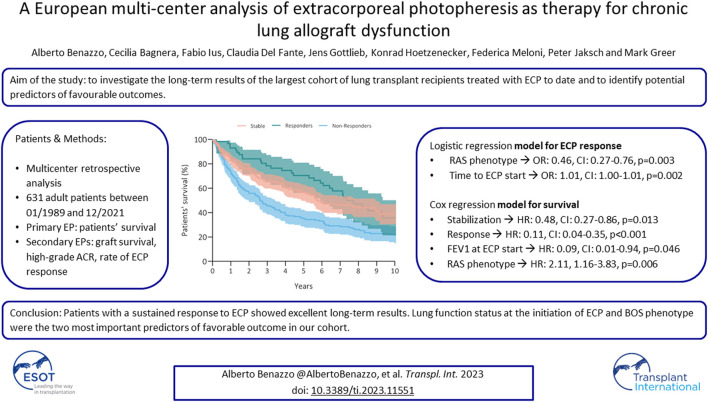

## Introduction

Chronic lung allograft dysfunction (CLAD) remains the major long-term cause of graft loss, affecting up to 60% of recipients within 5 years after lung transplantation (LTx) [1]. Although significant improvements have been implemented in the diagnosis and management of CLAD, effective treatment options are still lacking. Over the last two decades, extracorporeal photopheresis (ECP) has been increasingly used, to stabilize the deterioration of lung function besides other possible strategies, such as immunosuppression augmentation or administration of azithromycin [2–5]. ECP is an extracorporeal therapy, combining leukapheresis with photoactivation. It consists in the incubation of mononuclear cells with 8-methoxypsoralen (8-MOP) and subsequent activation of 8-MOP with ultraviolet A radiation. The cells are then reinfused into the patient. 8-MOP is a biologically inert substance, but in the presence of UVA light it cross-links DNA by forming covalent bonds with pyrimidine bases and causes cell apoptosis [6]. ECP has been firstly developed for treatment of cutaneous T cell lymphomas and later used in a variety of other indications including graft-versus-host disease and organ transplantation [7]. Up to date, only a limited number of LTx programs use ECP as a treatment for CLAD. To date published evidence is limited to single center retrospective analyses. According to available evidence, approximately 60%–70% of treated recipients profit from ECP, while in the rest of the treated patients lung function continues to deteriorate. In the current analysis, we examine the long-term outcomes of the largest cohort of lung transplant recipients treated with ECP to date.

## Methods

This is a retrospective multicenter analysis, including all lung transplant recipients transplanted between January 1989 and December 2021 and treated with ECP over the same time period in three European centers: Medical University of Vienna, Hannover Medical School Hannover and IRCCS Policlinico San Matteo. The primary endpoint was patient survival. Secondary endpoints were rate of ECP response, rate of high grade acute cellular rejection (ACR) and graft survival. Inclusion criteria were all patients ≥18 years, commencing ECP for progressing CLAD. This study has been approved by the Ethical Committe and was conducted according the declaration of Helsinki. The study was registered to *clinicaltrials.gov* with the number NCT04792294.

Spirometry was performed and interpreted according to ATS/ERS guidelines [8]. Values collected for the analysis were Forced Expired Volume in 1s (FEV1), Forced Vital Capacity (FVC) and Total Lung Capacity (TLC). Individual patient spirometry baselines were calculated based on the most recent ISHLT recommendations, with the mean value of the 2 best postoperative measurements obtained >3 weeks apart [9]. Diagnosis of CLAD was established by two independent physicians according to the consensus report of the ISHLT(9). CLAD was confirmed if FEV1 decline of ≥20% persisted for at least 3 months after exclusion or treatment of possible secondary causes, e.g., infections, acute rejection or extrapulmonary causes. Spirometry, TLC measurements and CT appearance were used to define CLAD phenotypes [9]. All transbronchial biopsies between transplantation and initiation of CLAD were included in the analysis and were classified according to ISHLT criteria [10]. A high-grade ACR was considered as A≥2, while high-grade LB was considered as B≥2.

Patient diagnosed with definite CLAD received a trial with azithromycin or montelukast for at least 3 months depending on center-specific clinical practice. In case of further deterioration, recipients started ECP. ECP was performed either on-line or off-line. On-line ECP was performed using the Therakos® CELLEX® Photopheresis System (Therakos UK Ltd., a Mallinckrodt Pharmaceuticals company), which is a closed-loop sterile system. The procedure has been described in detail elsewhere [11]. During ECP, peripheral blood mononuclear cells is separated from the whole blood in a Latham centrifuge (Latham International, Chesterton, UK) at 2,700 RPM. The collected cells (buffy-coat bag) is treated with 8-methoxypsoralen solution (UVADEX®, Therakos, Mallinckrodt Pharmaceuticals) and exposed extracorporeally to ultraviolet A light (1–2 J/m2) before reinfusion to the patient. During each treatment, four to six collection cycles are performed or 1,500 mL blood is processed, depending on the patient’s hematocrit level. Initially a 2 day treatment cycle was performed every second week for the first two to 6 months, according to institutional preferences. Then, a 2 day treatment cycle was performed once a month. When ECP was performed using the off-line technique, PBMCs were collected from the patient using a cell separator device, processing 1.5–2 blood volumes. Hemocytometric analysis was performed on the product at the end of each collection (quality control). Then, cells were irradiated (UV-A at 2 J/cmq; Macogenic, Macopharm a, France) after the dilution with saline solution and the addition of 8-methoxypsoralen (at 200 ng/mL concentration). Finally, the photoactivated PBMCs were immediately reinfused into the patient [12].

Responders were defined as patients with >10% improvement in FEV1 compared with the value at the time ECP treatment was started. Stable patients were defined as patients with ≤10% improvement or ≤10% worsening of FEV1 compared with the value at the time of initiation of ECP treatment. Non-responders were defined as patients who had a decline of >10% after ECP treatment. Interim response was evaluated at 3 and 6 months and long-term response was evaluated at completion of ECP or at the time of data analysis in the patients currently on ECP for >6 months. The rate of lung function decline was defined as a decrease in FEV1 in ml between two time points: positive values indicate a decrease in ml per month, whereas negative values indicate an increase in ml per month.

### Statistical Analysis

Categorical variables were reported as absolute and relative frequencies (%), continuous variables as median (interquartile range, IQR) or mean (± standard deviation). Relative frequencies were calculated based on the number of patients alive in follow-up at the respective timepoint. Chi-square tests, Fisher exact tests, Mann-Whitney U-tests, or ANOVA were used to compare variables as applicable. Survival curves were generated with the Kaplan-Meier method and compared by log-rank tests. Univariate and multivariable Cox regression were performed to find risk factors for mortality. Variables were included in a multivariable Cox regression when they reached the level of significance in the univariate analysis. Univariate and multivariable logistic regression were performed to find predictors of response (defined as stable and responders) to ECP. Variables were included in a multivariable logistic regression when they reached the level of significance in the univariate analysis. Data was analyzed using SPSS version 26.0 software or R 3.4.2 and graphics were designed with GraphPad Prism 6.

## Results

### Patients’ Demographics

This multicenter analysis included 631 patients from three European centers. Forty-eight percent (*n* = 291) were female and the mean age was 49 years (IQR: 35–56). The underlying diagnosis was COPD in 37% (*n* = 225) of patients, fibrosis in 25% (*n* = 155), iPAH in 10% (*n* = 57), and CF in 17% (*n* = 106). Twenty-three percent (*n* = 131) of patients had high-risk CMV mismatch, and 86% (*n* = 524) underwent bilateral lung transplantation. The median baseline FEV1 was 2.7 (2.1–3.9) L/min and the median baseline TLC was 5.5 L (4.7–6.5). Eighty-eight patients (18%) had high-grade ACR, and 74 (15%) had high-grade LB. Eighty-seven percent of patients treated with ECP had BOS patients, and only a minority (78, 13%) had RAS at the time of ECP initiation. The median time to CLAD after transplantation was 34 (18–64) months. Before initiation of ECP, 90% (*n* = 553) of patients had been treated with azithromycin and 36% (*n* = 221) with montelukast. Full demographic data can be found in Table 1.

**TABLE 1:** Patients’ characteristics.

Patients’ characteristics (N = 613)			
Female (n, %)			291, 48%
Age at LuTx (median, IQR)			49 (35–56)
High-risk CMV mismatch (n, %)			131, 23%
Underlying diagnosis		COPD (n, %)	225, 37%
		Fibrosis (n, %)	155, 25%
		iPAH (n, %)	57, 10%
		CF (n, %)	106, 17%
		CLAD (n, %)	31, 5%
		Others (n, %)	39, 6%
Type of Tx		DLuTX (n, %)	524, 86%
		SLuTX (n, %)	67, 11%
		HLuTx (n, %)	21 (3%)
FEV1 baseline (L/min) (median, IQR)			2.7 (2.1–3.9)
TLC baseline (L) (median, IQR)			5.5 (4.7–6.5)
High-grade ACR (n, %)			88, 18%
High-grade LB (n, %)			74, 15%
CLAD phenotypes		BOS (n, %)	513, 87%
		RAS (n, %)	78, 13%
Time to CLAD (months) (median, IQR)			34 (18–64)
Azithromycin (n, %)			553 (90%)
Montelukast (n, %)			221 (36%)
FEV1 at ECP start (L/min) (median, IQR)			1.4 (1.1–1.9)
TLC at ECP start (L) (median, IQR)			5.2 (4.1–6.2)
FEV1 at ECP (% baseline) (median, IQR)			56 (44–67)
Time to ECP (months) (median, IQR)			46 (26–88)
ECP cycles (median, IQR)			15 (11–25)
Response to 3 months of ECP	Stable (n, %)		319 (61%)
	Responder (n, %)		43 (8%)
	Non-Responder (n, %)		130 (25%)
	Death within 3 months (n, %)		32 (6%)
Response to 6 months of ECP	Stable (n, %)		294 (52%)
	Responder (n, %)		57 (10%)
	Non-Responder (n, %)		138 (24%)
	Death within 6 months (n, %)		79 (14%)
Long-term response to ECP	Stable (n, %)		252 (42%)
	Responder (n, %)		55 (9%)
	Non-Responder (n, %)		160 (26%)
	Death within 12 months (n, %)		138 (23%)

Abbreviations. N, numbers; IQR, interquartile range; SD, standard deviation; ECP, extracorporeal photopheresis; LuTx, lung transplantation; CMV, cytomegalovirus; COPD, chronic obstructive pulmonary disease; iPAH, idiopathic pulmonary arterial hypertension; CF, cystic fibrosis; CLAD, chronic lung allograft dysfunction; ReTx, retransplantation; DLuTx, double lung transplantation; SLuTx, single lung transplantation; HLuTx, heart-lung transplantation; ACR, acute cellular rejection; LB, lymphocytic bronchiolitis; BOS, bronchiolitis obliterans syndrome; RAS, restrictive allograft syndrome; ECP, extracorporeal photopheresis.

### Extracorporeal Photopheresis

Recipients with CLAD started ECP after a median of 34 months (18–64) after lung transplantation. The median FEV1 at the start of ECP was 1.4 L/min (1.1–1.9) and the median TLC was 5.2 L (4.1–6.2). ECP was performed for a median of 15 cycles (11–25). Response rate decreased at 3, 6 months and at the end of ECP. After 3 months of ECP, 61% showed stabilization of lung function, 8% showed an improvement and 25% showed a further worsening. Within the first 3 months of therapy, 6% of patients died. After 6 months of ECP, 52% exhibited stabilization of lung function, 10% improvement and 24% a worsening. Within the first 6 months of therapy, 14% of patients died. Long-term stabilization was achieved in 42%, improvement in 9%, and no response in 26%. Within the first 12 months of therapy, 23% of patients died. Long-term stable patients and responders were predominantly BOS patients (*p* = 0.005, Table 2) while non-responders were mostly RAS (*p* = 0.005, Table 2) and a shorter time to ECP start (*p* < 0.001, Table 2). A logistic regression was performed to find predictors of response to ECP (Table 3). Interestingly, in the multivariable regression model, RAS phenotype (OR: 0.46, CI: 0.27–0.76, *p* = 0.003) represented the only risk-factor for failed response while longer time to initiation of ECP (OR: 1.01, CI: 1.00–1.01, *p* = 0.002) seems to be predictive of a favorable response.

**TABLE 2:** Demographics per group.

		Stable (*n* = 252)	Responders (*n* = 55)	Non-Responders (*n* = 160)	Death within 12 months (*n* = 138)	*p*-value
Age at LuTx (median, IQR)		49 (34–56)	50 (39–56)	47 (30–54)	53 (37–59)	.030
High-risk CMV mismatch (n, %)		57 (23%)	14 (26%)	32 (21%)	38 (28%)	.579
Underlying diagnosis	COPD (n, %)	94 (37%)	23 (42%)	53 (33%)	53 (38%)	.943
	Fibrosis (n, %)	61 (24%)	14 (26%)	44 (28%)	35 (25%)	
	iPAH (n, %)	25 (10%)	4 (8%)	18 (11%)	9 (7%)	
	CF (n, %)	43 (17%)	6 (10%)	30 (19%)	24 (17%)	
	CLAD (n, %)	11 (5%)	4 (7%)	7 (4%)	9 (7%)	
	Others (n, %)	18 (7%)	4 (7%)	8 (5%)	8 (6%)	
Type of Tx	DLuTX (n, %)	213 (85%)	47 (85%)	131 (83%)	125 (91%)	.299
	SLuTX (n, %)	28 (11%)	8 (15%)	21 (13%)	10 (7%)	
	HLuTx (n, %)	11 (4%)	0	7 (4%)	3 (2%)	
FEV1 baseline (L/min) (median, IQR)		2.8 (2.2–3.4)	2.5 (2–3.2)	2.8 (2.3–3.4)	2.7 (2.2–3.1)	.101
TLC baseline (L) (median, IQR)		5.8 (4.7–6.9)	5.7 (4.8–6.5)	5.3 (4.7–6.4)	5.3 (4.4–6.2)	.056
Higher-grade ACR (n, %)		36 (17%)	3 (7%)	27 (22%)	22 (19%)	.181
Higher-grade LB (n, %)		27 (13%)	3 (7%)	23 (19%)	20 (17%)	.213
CLAD phenotypes	BOS (n, %)	**218 (92%)**	**49 (93%)**	**126 (81%)**	**113 (83%)**	**.005**
	RAS (n, %)	**20 (8%)**	**4 (7%)**	**30 (19%)**	**23 (17%)**	
Time to CLAD (months) (median, IQR)		35 (21–73)	37 (17–64)	34 (18–63)	30 (15–52)	.245
Azithromycin (n, %)		232 (93%)	51 (93%)	138 (87%)	125 (91%)	.208
Montelukast (n, %)		95 (38%)	20 (37%)	59 (37%)	47 (34%)	.914
FEV1 at ECP start (L/min) (median, IQR)		**1.6 (1.2–2)**	**1.2 (0.9–1.6)**	**1.5 (1.2–1.9)**	**1.3 (1–1.7)**	**<.001**
TLC at ECP start (L) (median, IQR)		5.3 (4.4–6.5)	6.1 (5.3–7.1)	4.8 (3.8–6)	5.1 (3.8–6)	.138
FEV1 at ECP (% baseline) (median, IQR)		60 (48–70)	52 (42–58)	55 (45–68)	49 (39–62)	.074
Time to ECP (months) (median, IQR)		**56 (33–101)**	**44 (24–92)**	**41 (24–78)**	**39 (22–76)**	**<.001**
Rate of FEV1 decline before ECP (mL/month) (median, IQR)		**18 (10–35)**	**24 (12–57)**	**28 (14–51)**	**29 (13–60)**	**<.001**
Rate of FEV1 decline in 3 months of ECP (mL/month) (median, IQR)		**10 (-27–43)**	**−113 (-160–-37)**	**57 (10–120)**	**72 (23–137)**	**<.001**
Rate of FEV1 decline in 6 months of ECP (mL/month) (median, IQR)		**4 (-10–23)**	**−65 (-107–-8)**	**36 (16–77)**	**43 (20–82)**	**<.001**

The rate of FEV1 decline was calculated as the difference in ml between two time points per month: positive values indicate a decline in ml per month, while negative values indicate an increase in ml per month. Abbreviations. N, numbers; IQR, interquartile range; SD, standard deviation; LuTx, lung transplantation; CMV, cytomegalovirus; COPD, chronic obstructive pulmonary disease; iPAH, idiopathic pulmonary arterial hypertension; CF, cystic fibrosis; CLAD, chronic lung allograft dysfunction; ReTx, retransplantation; DLuTx, double lung transplantation; SLuTx, single lung transplantation; HLuTx, heart-lung transplantation; ACR, acute cellular rejection; LB, lymphocytic bronchiolitis; BOS, bronchiolitis obliterans syndrome; RAS, restrictive allograft syndrome; ECP, extracorporeal photopheresis.

Bold values are the significant results.

**TABLE 3:** Logistic regression for response to ECP.

		OR (CI)	*p*-value	Adjusted OR (CI)	*p*-value
Age at LuTx		1.00 (0.99–1.02)	.574		
High-risk CMV mismatch		1.06 (0.71–1.58)	.771		
Underlying diagnosis	COPD	Reference			
	Fibrosis	0.74 (0.48–1.33)	.166		
	iPAH	0.93 (0.50–1.72)	.821		
	CF	0.88 (0.54–1.44)	.615		
	CLAD	1.09 (0.49–2.43)	.839		
	Others	0.99 (0.48–2.06)	.990		
Type of Tx	DLuTX	Reference			
	SLuTX	0.89 (0.53–1.51)	.673		
	HLuTx	1.13 (0.45–2.86)	.791		
FEV1 baseline (L/min)		1.01 (0.83–1.22)	.937		
TLC baseline (L)		0.99 (0.99–1.00)	.713		
Higher-grade ACR		0.86 (0.53–1.39)	.543		
Higher-grade LB		0.62 (0.37–1.02)	.062		
CLAD phenotypes	BOS	Reference		Reference	
	RAS	**0.45 (0.27–0.73)**	**<0.001**	**0.46 (0.27–0.76)**	**.003**
Time to CLAD (months)		1.00 (0.99–1.01)	.119		
Azithromycin		**1.77 (1.01–3.12)**	**.049**	1.47 (0.81–2.66)	.207
Montelukast		1.05 (0.74–1.49)	.778		
FEV1 at ECP start (L/min)		1.08 (0.83–1.41)	.562		
TLC at ECP start (L)		1.36 (0.80–1.71)	.100		
FEV1 at ECP (% baseline)		1.02 (0.99–1.01)	.668		
Time to ECP (months)		**1.01 (1.00–1.10)**	**<0.001**	**1.01 (1.00–1.01)**	**.002**

Bold values are the significant results.

### Outcomes

**FIGURE 1:**
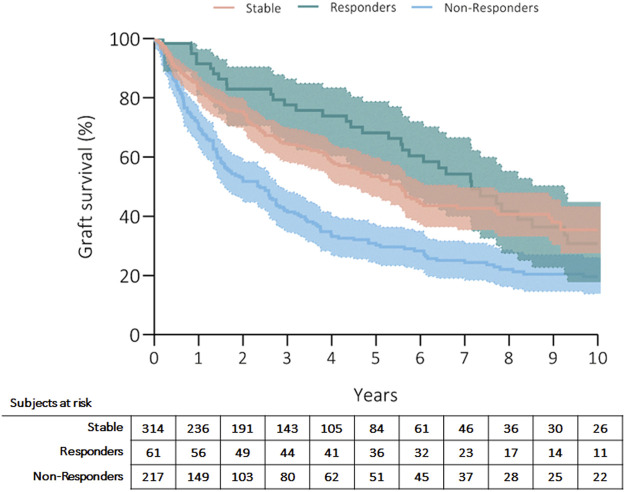
Kaplan Meier’s curve showing patients’ survival after initiation of ECP. Curves have been compared with log-rank test.

**FIGURE 2:**
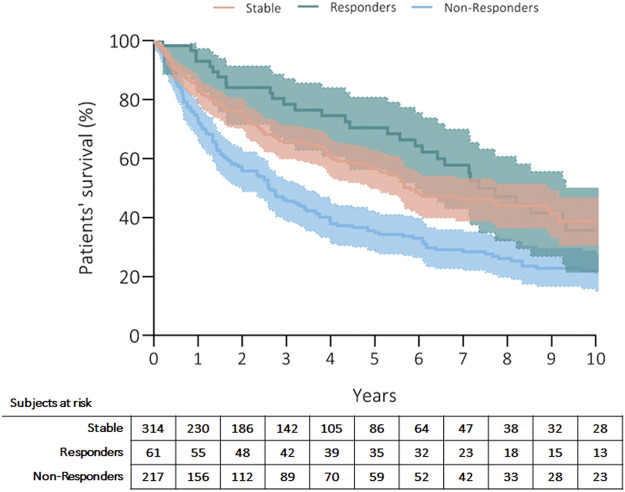
Kaplan Meier’s curve showing overall graft survival after initiation of ECP. Curves have been compared with log-rank test.

Three hundred fifty patients (57%) died, and the most common causes of death were CLAD in 47% (*n* = 164, 27% from the whole cohort) and sepsis in 19% (*n* = 66, 11% from the whole cohort) of recipients (Table 4). Patients’ survival rates after initiation of ECP were at 5 years: 56% in stable, 70% in responders and 35% in non-responders; at 10 years: 39% in stable, 36% in responders and 23% in non-responders (*p* = 0.001) (Figure 1). Fifty-three patients (9%) received retransplantation. Graft survival rates after initiation of ECP were at 5 years: 53% in stable, 68% in responders and 30% in non-responders; at 10 years: 35% in stable, 31% in responders and 20% in non-responders (*p* = 0.001) (Figure 2).

**TABLE 4:** Outcomes.

**Outcomes**		
**ReTx (n, %)**		**53 (9%)**
Death (n, %)	All	350 (57%)
	CLAD (n, %)	164 (47%)
	Sepsis (n, %)	66 (19%)
	Malignancy (n, %)	22 (6%)
	Others (n, %)	98 (28%)
Graft survival (months) (median, IQR)		98 (53–152)

Abbreviations. N, numbers; IQR, interquartile range; ReTx, retransplantation; CLAD, chronic lung allograft dysfunction.

Cox regression was performed to examine the effect of response to extracorporeal photopheresis on patient survival after adjusting for confounding factors (Table 5). Multivariable regression showed that long-term stabilization (HR: 0.48, CI: 0.27–0.86, *p* = 0.013) or response (HR: 0.11, CI: 0.04–0.35, *p* < 0.001) to ECP were associated with survival. Interestingly, absolute FEV1 at baseline ECP was also protective (HR: 0.09, CI: 0.01–0.94, *p* = 0.046). RAS phenotype was the only risk factor for mortality (HR: 2.11, 1.16–3.83, *p* = 0.006).

**TABLE 5:** Cox Regression for patient survival.

			HR (CI)	*p*-value	Adjusted HR (CI)	*p*-value
Age at LuTx			**1.03 (1.02–1.04)**	**<.001**	1.02 (0.99–1.05)	.214
High-risk CMV mismatch			1.18 (0.92–1.53)	.198		
Underlying diagnosis	COPD		Reference			
	Fibrosis		1.03 (0.79–1.34)	.828		
	iPAH		**0.51 (0.34–0.77)**	**<.001**	0.90 (0.24–3.38)	.886
	CF		0.72 (0.53–1.01)	.054		
	CLAD		0.88 (0.54–1.45)	.617		
	Others		0.91 (0.57–1.44)	.684		
Type of Tx	DLuTX		Reference			
	SLuTX		0.81 (0.59–1.12)	.199		
	HLuTx		0.50 (0.27–1.11)	.124		
FEV1 baseline (L/min)			**0.87 (0.76–0.98)**	**.023**	1.92 (0.65–5.62)	.237
TLC baseline (L)			1.00 (0.99–1.01)	.560		
Higher-grade ACR			0.91 (0.66–1.26)	.577		
Higher-grade LB			1.35 (0.98–1.86)	.064		
CLAD phenotypes	BOS		Reference		Reference	
	RAS		**2.01 (1.53–2.63)**	**<0.001**	**2.11 (1.16–3.83)**	**.015**
Time to CLAD (months)			**0.99 (0.98–0.99)**	**<0.001**	0.99 (0.97–1.01)	.379
Azithromycin			0.71 (0.51–1.00)	.051		
Montelukast			0.94 (0.76–1.18)	.610		
FEV1 at ECP start (L/min)			**0.64 (0.53–0.76)**	**<0.001**	**0.09 (0.01–0.94)**	**.046**
TLC at ECP start (L)			**0.88 (0.78–0.99)**	**.029**	1.09 (0.95–1.27)	.227
FEV1 at ECP (% baseline)			**0.99 (0.98–0.99)**	**<0.001**	1.04 (0.98–1.11)	.187
Time to ECP (months)			**0.99 (0.98–0.99)**	**<0.001**	0.99 (0.97–1.01)	.064
Response to ECP at end of ECP		Stable	0.50 (0.40–0.63)	**<0.001**	**0.48 (0.27–0.86)**	**.013**
		Responder	0.48 (0.33–0.71)	**<0.001**	**0.11 (0.04–0.35)**	**<0.001**
		Non-Responder	Reference		Reference	

Bold values are the significant results.

## Discussion

Chronic lung allograft dysfunction remains the leading cause of morbidity and mortality after lung transplantation. According to the international benchmarks, median survival after the diagnosis of CLAD ranges between 3 and 5 years. Curative treatments have not been established yet, however, different therapeutic interventions can slow down the progression of allograft dysfunction. Extracorporeal photopheresis is an immunomodulatory therapy, which targets T-cell mediated injury and improves mortality and morbidity in a range of T-cell mediated diseases as well as graft-versus-host disease [13]. With the same rationale, ECP was introduced in solid organ transplantation as a salvage therapy for a range of indications. The current study, including more than 600 patients, presents the largest experience with ECP in a CLAD population to date. The herein reported results show that 63% of CLAD patients experienced a stabilization or improvement of the allograft function after ECP initiation, which was associated with a survival benefit.

To date, ECP treatment has been used as second-line therapy for CLAD after lung transplantation. However, efficacy data is based only on small single-center studies. Greer et al. found that RAS patients, as well as patients whose lung function deteriorated rapidly, had lower response rates and worse long-term outcomes [4]. Similarly, in another analysis, only BOS was associated with better outcomes [3]. A prospective study published by the Vienna group confirmed the results of previous retrospective analyses, showing a 61% response rate and improved survival in the responder population [5]. Recently, the Hannover group proposed an innovative approach to assessing CLAD patient outcomes using a temporal characterization of allograft function [14]. In this study, the authors not only reported a response rate to ECP comparable to previously published studies, but also suggested that grafts with lower performance at the beginning of ECP were more likely to be associated with worse outcomes [14]. The current analysis includes 631 patients from three European centers with a long-standing experience with ECP. Long-term stabilization of graft function could be achieved in 53% of the cohort, 10% showed an improvement while the remaining 37% fail to respond to ECP. These rates confirm previously published experience. Our data showed that the BOS phenotype was associated with a higher response rate and improved survival, while RAS phenotype was associated with lower response rate and higher mortality. Interestingly, absolute TLC at initiation of ECP did not seem to be a risk factor. Thus, the results of our analysis suggests that the unresponsiveness of this subpopulation is related more to the restrictive phenotype *per se* and its underlying pathophysiology than to the reduction of lung volumes. This finding is not completely novel. Indeed, the majority of previous series could show the same difference in response between the phenotypes [3, 5, 14], however, the mechanistic reason remains elusive. RAS is characterized by a more intense allogeneic inflammatory response followed by diffuse fibrotic processes in various anatomic compartments [15]. The most widely accepted hypothesis is that a severe and fulminant immune response is triggered by an acute event such as ACR, AMR, or viral infection, which initiates extensive pro-fibrotic events involving airways, pleura, septum, alveoli, and vessels [15]. On the other side, BOS is mostly a chronic airway-centered disease. External exposures, airway-specific autoantibodies, a type 17 immune response, and early ischemic injury to the airway epithelium can chronically affect lung allografts via the airway [15]. It is reasonable to speculate that the slowly evolving immunomodulatory effect of ECP is more effective in the subclinical injury typical of BOS. In addition, there is a hypothesis that ECP is less effective in modulating endothelial activation and fibrogenic mechanisms characteristic of RAS. Although RAS appears to be associated with CLAD progression and nonresponse to ECP, this alone can hardly explain the 37% nonresponse rate. Therefore, the mechanisms of action of ECP need to be further elucidated to understand its application and limitations in CLAD.

An important finding of the current study is that the absolute FEV1 value at the initiation of ECP is an independent predictor of survival in our cohort but unrelated to ECP treatment response. This is a novel finding is new and suggests that the use of baseline lung function estimates may be misleading in the design of clinical trials intended to assess functional response to new CLAD therapies. Indeed, the risk of baseline estimates is that they tend to overestimate lung allograft function, thereby discriminating against patients at higher risk for worse outcomes. As early as 2007, Burton et al warned that the use of an estimated baseline FEV1 represents a statistical bias and disadvantages recipients with lower baseline values [16]. Applied to the current topic, this means that patients with lower absolute FEV1 values are classified as having a more severe CLAD grade, while also having poorer functional reserve. As a result, they deteriorate more rapidly and, in most cases, end in fatal respiratory failure before they can experience a benefit from the started therapy. The prospective nonrandomized study conducted by the EPI Study Group is the best example of this limitation in the context of ECP(17). Because of the study design, patients with low FEV1 values and more rapid deterioration were more likely to undergo ECP and have a fatal outcome [17]. On the other hand, however, ECP was associated with a 93% reduction in FEV1 decline, and none of the fatal outcomes were related to ECP(17). Instead, 92% of mortality cases were due to end-stage lung failure. Similar results were observed in the recent work from Hannover, which showed that absolute FEV1 at the onset of ECP had the greatest impact on patient and graft survival [14]. Taken together, this underscores that the absolute FEV1 at the initiation of ECP may be the most important confounding bias in evaluating outcomes over time and, in parallel, this finding suggests that ECP should be initiated at earlier stages rather than used as rescue therapy when functional reserve has reached a dangerous level.

Another important observation of this study is that a longer interval to initiation of ECP is associated with better outcomes. This is clearly a surrogate measure of the severity of CLAD. Patients with a shorter time to initiation of ECP were those whose condition deteriorated rapidly and who had a more fulminant course. In these patients, the ECP effect may never have manifested. Similar findings were already observed in smaller single-center series [3, 5]. Moreover, it is already known that the effects of ECP are not apparent for at least 4–5 months in GvHD patients and over 12 months in scleroderma patients. Therefore, in conjunction with the previously discussed findings, possible use of ECP could be considered to increase the efficacy of this therapy in CLAD patients.

We are aware that this study is not free of limitations. First, because of the retrospective nature of the study, there is a possibility that the data were miscoded. In addition, we cannot exclude the possibility that the indication for ECP became more liberal over time because of increasing clinical experience. Another limitation arises from the multicenter nature of the study, as clinical practice might differ among the centers. Another limitation of our retrospective multicenter analysis is that data on AMR and DSAs are not included. Because the pathogenic role of AMR and DSA in lung transplantation is relatively recent and the analysis covers a period of more than 20 years, these data are available only for patients treated in the last 5 years. Finally, the three centers use different ECP systems, which could potentially affect the results.

Despite these limitations, this study provides the long-term outcomes of ECP application in CLAD patients in the largest published cohort to date. Two thirds of the cohort had a sustained response to ECP, showing excellent long-term results in CLAD patients compared to international benchmarks of untreated patients. Lung function status at the initiation of ECP and BOS phenotype were the two most important predictors of favorable outcome in our cohort. Both the excellent results and the new evidence support this therapy and suggest that early initiation of ECP may be beneficial in terms of both response and survival. Further studies are needed to elucidate the exact mechanisms of action and thus improve its application.

## Data Availability

The data analyzed in this study is subject to the following licenses/restrictions: The dataset are property of the Medical University of Vienna, Hannover Medical School and IRCCS Policlinico San Matteo. Access to the dataset can be provided after formal approval of the legal departments of the three involved centers and the of the first and last authors. Requests to access these datasets should be directed to rechtsabteilung@meduniwien.ac.at.
